# Structural development and physicochemical properties of starch in caryopsis of super rice with different types of panicle

**DOI:** 10.1186/s12870-019-2101-7

**Published:** 2019-11-08

**Authors:** Xinyu Chen, Mingxin Chen, Guoqiang Lin, Yang Yang, Xurun Yu, Yunfei Wu, Fei Xiong

**Affiliations:** Jiangsu Key Laboratory of Crop Genetics and Physiology/Co-Innovation Center for Modern Production Technology of Grain Crops/Joint International Research Laboratory of Agriculture & Agri-Product Safety of the Ministry of Education/College of Bioscience and Biotechnology, Yangzhou University, 48 Wenhui East Road, Yangzhou, 225009 China

**Keywords:** Rice, Caryopsis, Amyloplast, Physicochemical properties

## Abstract

**Background:**

Starch is the main storage substance in rice caryopsis and its properties will determine the quality of rice. Super rice has been extensively studied due to its high-yield characteristics, but the knowledge of amyloplast development and starch quality in caryopsis of super rice especially with large panicle is limited.

**Results:**

To address this, large panicle typed and normal panicle typed super rice cultivar Yongyou2640 (YY2640) and Nangeng9108 (NG9108) were investigated in this study. The development of amyloplast in YY2640 caryopsis was better than NG9108, showing faster degradation rate of pericarp amyloplast and better filling degree of endosperm amyloplast. Meanwhile, the starch granule of YY2640 presented as polyhedral shape with smooth surface and the granule size was slightly larger than NG9108. The starch of YY2640 exhibited the lower amylose content, ratio of amylose to amylopectin and the higher level of amylopectin short and long branch-chains compared with NG9108, but there was no significant difference in amylopectin branching degree between them. Two rice starches both showed the characteristics of A-type crystal, and the relative crystallinity and external ordered degree of YY2640 starch were higher than those of NG9108. Furthermore, YY2640 starch showed better pasting properties with lower pasting temperature, shorter pasting time, higher peak viscosity, trough viscosity, breakdown value and lower setback value because of lower apparent amylose content.

**Conclusions:**

Overall, the development and filling of amyloplast in YY2640 caryopsis were better than those of NG9108, thus leading to better starch quality of YY2640.

## Background

Rice (*Oryza sativa* L.) is one of the most important cereal crops in the world, whose production ranks third, second only to maize and wheat. About 50% of global population consume rice as staple food and more than 20 million people take in energy and nutrition from rice and its by-products [1]. Especially in Asia, including China, rice plays a pivotal role in food production [2]. However, the pressure on yield increase of crops is increasing with the rapid population growth, especially in rice production [3]. Therefore, achieving super high yield of rice is of great significance for ensuring food security and promoting social stability. To this end, how to further improve rice yield and quality has become the focus of rice researchers and many countries also regard high yield as the main target of rice breeding. Since 1996, the Ministry of Agriculture of China has also officially launched the super rice research and it has succeeded in cultivating the new rice varieties with high yield potential and good quality by using intersubspecific heterosis and improvement plant type design [4, 5]. For example, the Yongyou series is a new cultivated super hybrid rice variety with the characteristics of large panicle, good agronomic traits and high yield which has been widely promoted in recent years [6, 7].

Compared with conventional rice cultivars, super rice can significantly increase yield by 15–20% in field trials and by about 10% in large-area production [8]. Previous studies showed that many agronomic and physiological traits of super rice are important factors contributed to high grain yield [9]. Huang et al. found that the source activity of super rice which is characterized with leaf area index, leaf area duration, photosynthetic rate was greater than that of check rice cultivar [10]. Also, Zhang et al. pointed out that the biomass, oxidative activity, zeatin content and zeatin nucleosides content of super rice roots were higher, which resulted in larger sink size of aerial part and ultimately led to high yield [11].

Caryopsis is an important nutrient storage organ of rice, including seed coat, pericarp, endosperm and embryo. Starch, as the main storage substance of rice caryopsis, accounts for about 80% of the total grain weight and it is composed of amylose and amylopectin which form crystal called starch granule in a certain ratio [12]. In rice caryopsis, starch accumulates in the form of compound starch granules in endosperm amyloplast. As the development of caryopsis, the number and volume of amyloplast continue to increase [13, 14]. The development and filling of amyloplast in endosperm cells, as well as the properties of starch, can influence the yield and quality of rice and determine the use of rice. With the improvement of living standards, the quality of rice has attracted more attention. The properties of rice starch play an important role in determining rice nutrition and cooking quality, so there are many studies on this field [15–17]. Wang et al. investigated the differences in structures and physicochemical properties of starch from six wild rice varieties [18]. Additionally, Zhu et al. thought that rice with high amylose content had poor cooking quality, while rice with low amylose content usually had good eating quality [19].

In recent years, the cultivation and application of super rice have significantly increased the yield of rice. The predecessors have done much work concerning the characteristics of super rice, the formation rule of super high yield, the cultivation techniques of super rice and so on. Wu et al. studied the synergistic evolution rule of grain yield components and the characters of super-high yielding japonica super rice [20]. Ma and Tao proposed a new cultivation technology in hybrid rice and revealed its high-yielding mechanism [21]. However, as far as we know, there are few studies on super rice starch and its quality, especially the knowledge on development of amyloplast and physicochemical properties of starch in caryopsis of large panicle typed super high yield rice is little known. In this study, super rice cultivar with large panicle Yongyou2640 (YY2640) and super rice cultivar with normal panicle Nangeng9108 (NG9108) were selected as materials. The morphology of caryopsis development was photographed and the structure development of amyloplast in pericarp and endosperm was observed. Meanwhile the physicochemical properties such as morphology, structure and pasting properties of starch derived from rice caryopsis were determined. The results will not only enrich the research of rice starch and deepen the study of super rice quality, but also provide an important reference for the processing and utilization of super rice starch and lay a foundation for better application of super rice.

## Results

### Development of caryopsis and panicle traits of mature rice

**Fig. 1:**
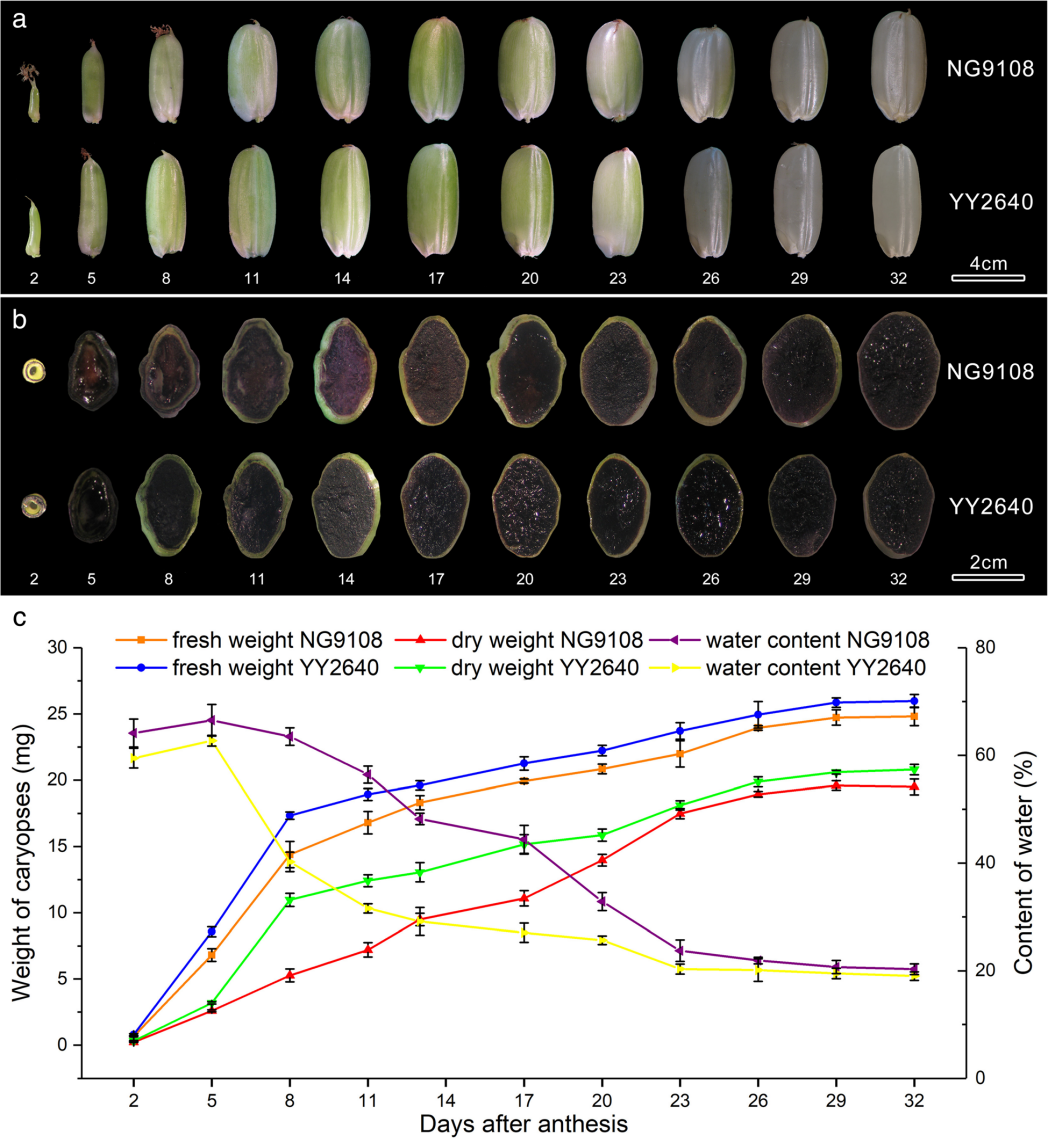
Changes in morphology and substance accumulation during caryopsis development in two rice cultivars. **a** profile of growing caryopsis; (**b**) I_2_/KI staining of growing caryopsis transverse sections; (**c**) dynamic changes of growing caryopsis fresh weight, dry weight and water content. Numbers on the bottom of subpanels (**b**, **c**) represent days of caryopsis development. Scale bar: (**a**) 4 cm, (**b**) 2 cm

At early stage of development, the caryopsis continued to expand and mainly grew longitudinally. The length of caryopsis was basically fixed at 8 days after anthesis (DAA), which was close to the length of mature caryopsis. At the same time, the caryopsis grew in the lateral direction and gradually became wider. At 14 DAA, the width of caryopsis tended to be stable and then the volume of caryopsis increased slowly. At 17–20 DAA, the caryopsis was basically shaped and its morphology and size were no longer changed afterwards. The pericarp of rice caryopsis was greenish at early development stage and then faded with the duration of grain filling. At 23 DAA, the pericarp of the caryopsis was mostly waxy white and only the dorsal of caryopsis remained green. At full ripening period, the caryopsis was dehydrated and hardened, showing a transparent state (Fig. 1a). The results of I_2_/KI staining of caryopsis transection were also observed (Fig. 1b). At the beginning of grain filling, there was no starch accumulation in endosperm tissue. So the endosperm was not stained by I_2_/KI but the pericarp was dyed black. At 5 DAA, starch began to accumulate in endosperm and increased rapidly. Meanwhile, the accumulation of starch in pericarp peaked and pericarp was stained black by I_2_/KI, as well as endosperm. As the development of caryopsis, the endosperm was continuously filled and the dyed endosperm became darker, while the pericarp got thinner and the color of pericarp stained by I_2_/KI gradually disappeared. These results indicated that the accumulation of endosperm starch continuously increased but pericarp starch firstly accumulated and then gradually degraded. The development of the two rice caryopsis was basically similar but there were significant differences in the morphology of caryopsis. The shape of NG9108 caryopsis was short and round, while the shape of YY2640 caryopsis was slender. Compared with NG9108, the YY2640 caryopsis developed faster and matured earlier with shorter grain filling time. In addition, the I_2_/KI staining degree of YY2640 caryopsis transection was deeper than that of NG9108, indicating more starch accumulation in YY2640 endosperm.

The dynamic changes in fresh weight, dry weight and water content of caryopsis during the growth cycle were also determined and the results are shown in Fig. 1c. The caryopsis of two rice cultivars both grew fast at early stage and grew slow at later stage. At 2–8 DAA, the increase rate of caryopsis fresh and dry weight was the fastest and subsequently slowed down. The fresh and dry weight of caryopsis reached the maximum at about 26 DAA and then got stable. The water content showed a downward trend during the development of caryopsis. At 5 DAA, the water content of the two rice caryopsis was the maximum and decreased rapidly subsequently. The decrease rate of water content tended to be flat at 23 DAA and eventually the water content was stable at about 20%. During the whole development of caryopsis, the fresh and dry weight of YY2640 were both higher than those of NG9108, while the water content was lower than NG9108. Additionally, the panicle length, panicle weight, grain number per panicle, seed setting rate of YY2640 were significantly higher than those of NG9108 and the 1000-grain weight of mature caryopsis also showed a higher level (Table 1).

**Table 1:** Panicle traits and 1000-grain weight of two rice cultivars

cultivar	main panicle length (cm)	main panicle weight (g)	grain number per panicle	seed setting rate (%)	1000-grain weight (g)
NG9108	17.40 ± 0.51	3.49 ± 0.05	205.00 ± 3.61	79.62 ± 2.35	17.33 ± 0.15
YY2640	23.63 ± 0.35^*^	7.45 ± 0.42^*^	381.67 ± 13.35^*^	88.03 ± 0.34^*^	18.09 ± 0.15^*^

Data are shown as means ± standard error, *n* = 3. For each column, asterisks indicate significant difference between rice cultivars at *p* < 0.05 as determined by t-test

From the results above, it can be concluded that the caryopsis of YY2640 was an elongated type and its morphological development process was basically similar to NG9108. However, YY2640 had many advantages in agronomic traits compared with NG9108, with faster caryopsis development process, more starch accumulation in endosperm, higher grain weight and better panicle traits, which might lead to the high yield.

### Development of amyloplast in pericarp and endosperm

**Fig. 2:**
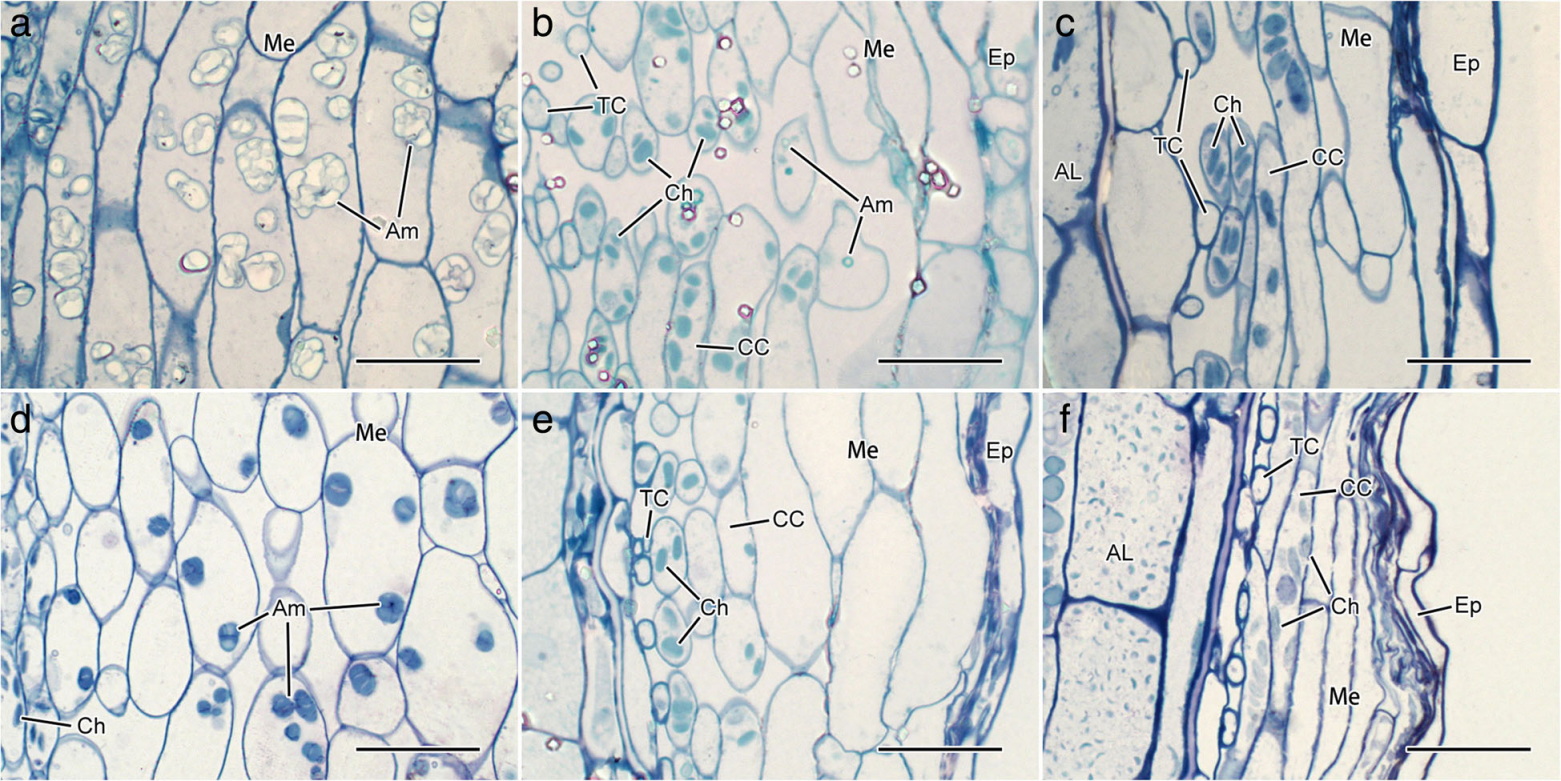
Development of pericarp amyloplast in two rice caryopsis. **a**-**c** microstructure of NG9108 caryopsis pericarp at 5, 10, 20 DAA; (**d**-**f**) microstructure of YY2640 caryopsis pericarp at 5, 10, 20 DAA. AL, aleurone layer; Am, amyloplast; CC, cross cell; Ch, chloroplast; Ep, epicarp; Me, mesocarp; TC, tube cell. Scale bar: 20 μm

Similar to wheat, the development of rice pericarp has undergone the process of occurrence, development and apoptosis with the growth of caryopsis [22]. Pericarp amyloplast mainly accumulates in mesocarp, which is spherical and contains numerous starch granules. The development of amyloplast shows a dynamic trend of synthesis-accumulation-degradation with the growth of pericarp [23]. At early development stage, the amyloplast rapidly accumulates and continuously expands in mesocarp cells. Afterwards, the amyloplast begins to degrade and disappears as the growth of pericarp. At 5 DAA, the apoptosis of mesocarp cells in YY2640 caryopsis was observed and the intercellular space was large. A large amount of pericarp amyloplast degraded and only slight amyloplast could be observed in mesocarp cells. Conversely, the cellular structure of mesocarp in NG9108 caryopsis was compact and the number of amyloplast in mesocarp cells was large (Fig. 2a, d). The statistical results also showed that the numbers and relative areas of amyloplast in the pericarp of YY2640 caryopsis were significantly lower than those of NG9108 (Fig. 4a, b). These indicated that the apoptosis of pericarp cells appeared earlier and the degradation of pericarp amyloplast was faster in YY2640 caryopsis. At 10 DAA, the pericarp became thinner and the pericarp amyloplast was almost degraded. There was only a small amount of small amyloplast in the pericarp of NG9108 while it was difficult to observe the residual amyloplast in the pericarp of YY2640 caryopsis (Fig. 2b, e). Also, the numbers and relative areas of amyloplast in pericarp cells of YY2640 were still lower than those of NG9108. At 20 DAA, the pericarp amyloplast disappeared and the pericarp cells dehydrated and shrunk in two rice caryopsis (Fig. 2c, f). The chloroplasts were still observed in the cross cells of endocarp, more of which were accumulated in the pericarp of NG9108. This may lead to the longer caryopsis development period compared with YY2640. All the results above manifested that the growth period of pericarp in YY2640 caryopsis was shorter, presenting as the faster apoptosis rate of pericarp cells and degradation rate of amyloplast.

**Fig. 3:**
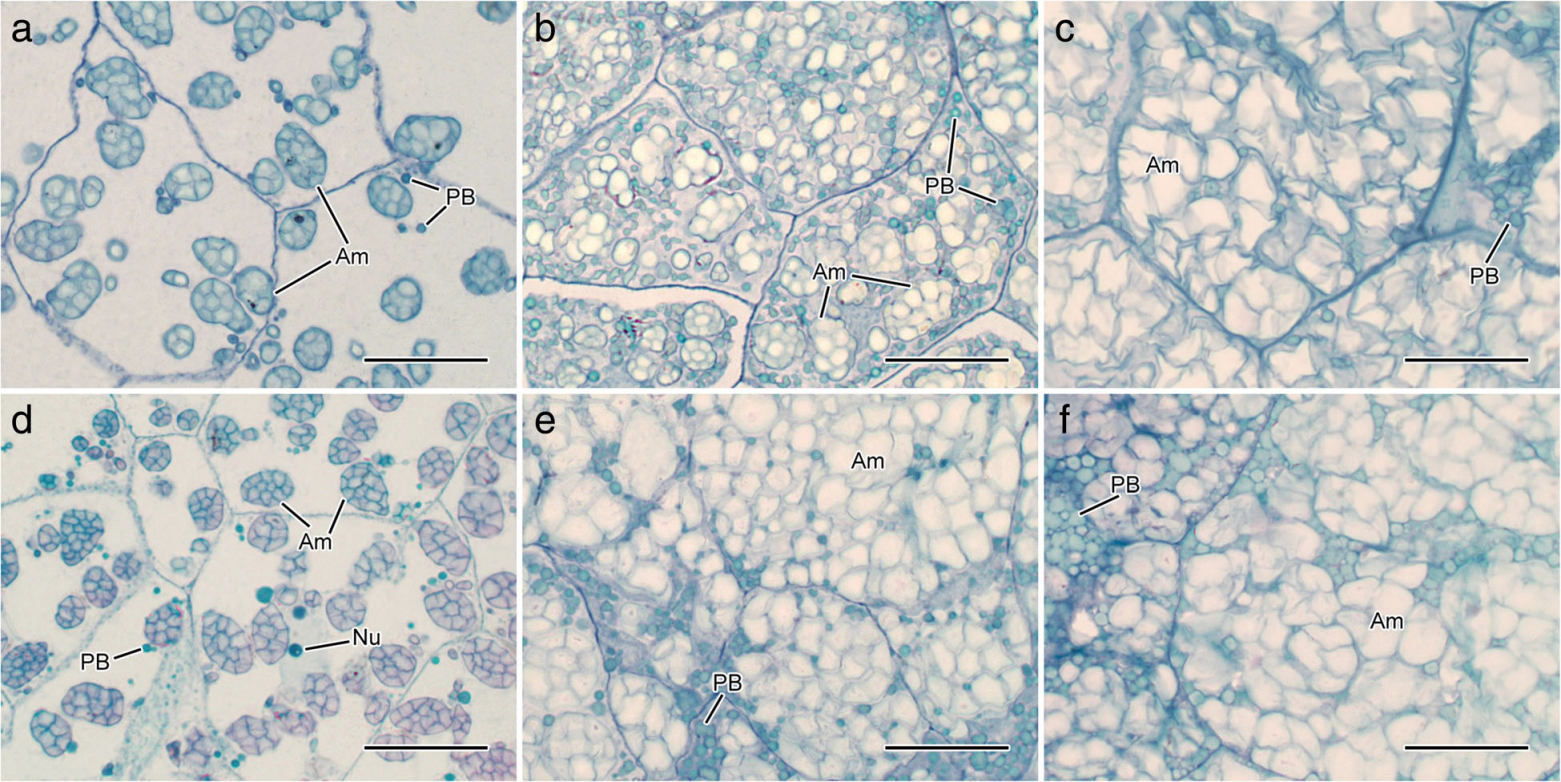
Development of endosperm amyloplast in two rice caryopsis. **a**-**c** microstructure of NG9108 caryopsis endosperm at 5, 10, 20 DAA; (**d**-**f**) microstructure of YY2640 caryopsis endosperm at 5, 10, 20 DAA. Am, amyloplast; PB, protein bodies. Scale bar: 20 μm

**Fig. 4:**
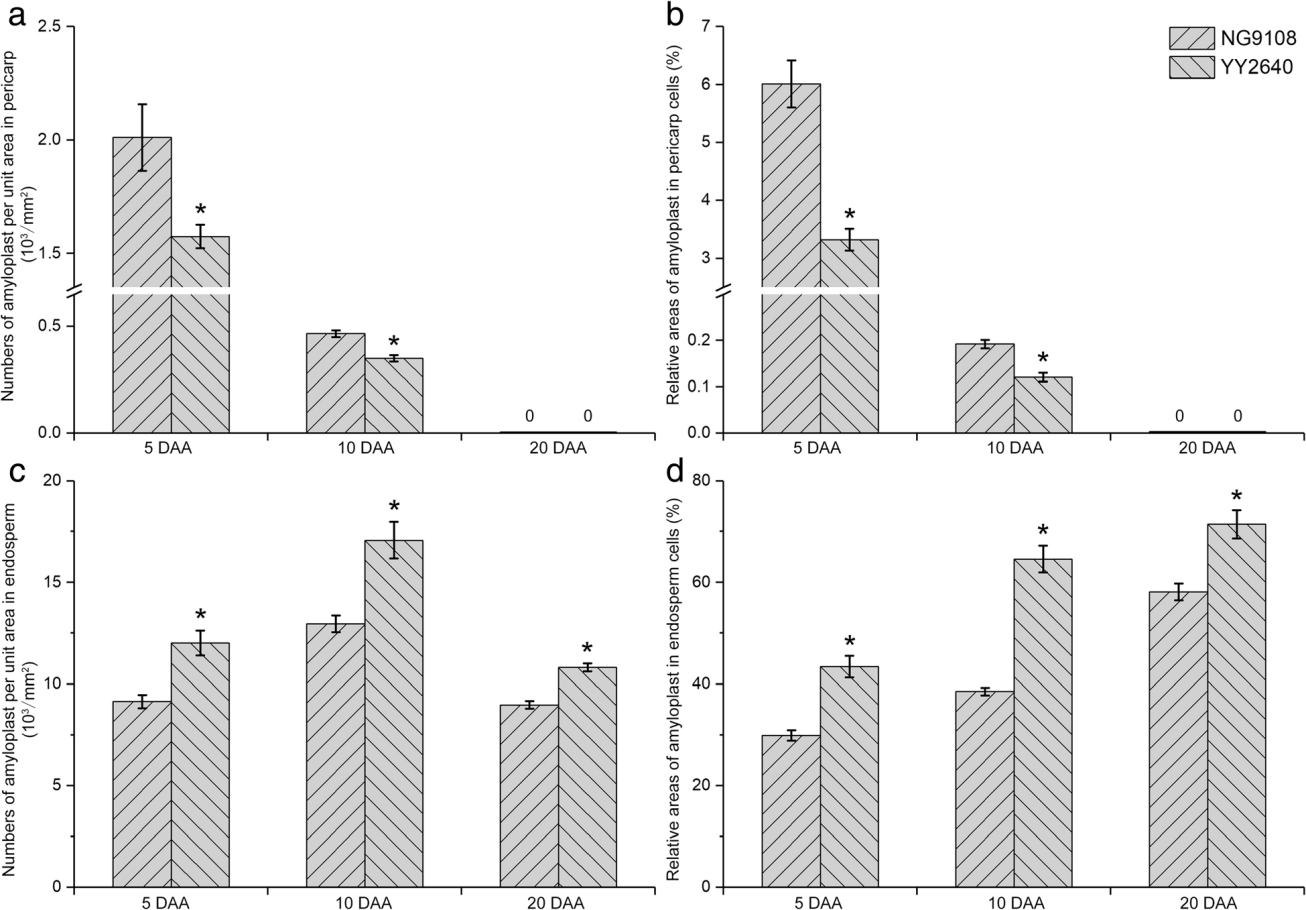
Accumulation characteristic of caryopsis amyloplast in two rice cultivars. **a** numbers of amyloplast per unit area in pericarp cells; (**b**) relative areas of amyloplast in pericarp cells; (**c**) numbers of amyloplast per unit area in endosperm cells; (**d**) relative areas of amyloplast in endosperm cells. Asterisks above the histogram indicate significant difference between rice cultivars at *p* < 0.05 (t-test)

Endosperm is the main component of rice caryopsis and its development process includes the division, growth and differentiation of endosperm cells and the filling of storage substance (mainly starch) [24]. As the development of caryopsis, the number and volume of endosperm amyloplast continue to increase until filling the entire endosperm [25]. At 5 DAA, there was some spherical or ellipsoidal amyloplast accumulating in the endosperm cells with several small protein bodies around (Fig. 3a, d). Compared with NG9108, there was more amyloplast accumulating in the endosperm of YY2640 caryopsis. At 10 DAA, the endosperm was further filled and the number and size of amyloplast increased. In NG9108 endosperm, the filling degree of amyloplast was not so well and large gaps between amyloplast were observed in which some small protein bodies accumulated (Fig. 3b). The amyloplast compactly filled in the endosperm of YY2640 was polyhedral and bulky and the aggregation of small protein bodies was observed in the gaps of amyloplast (Fig. 3e). At 20 DAA, the endosperm was completely filled with amyloplast which was squeezed each other and deformed. The granules in amyloplast were extruded into the shape of polyhedral (Fig. 3c, f). Moreover, the numbers of amyloplast per unit area and the relative areas of amyloplast in the endosperm of YY2640 were significantly higher than those of NG9108 during whole caryopsis development (Fig. 4c, d). These results all indicated that the development and filling of endosperm amyloplast of YY2640 were better than those of NG9108.

Based on the above results, it was concluded that the development of amyloplast in YY2640 caryopsis was better than NG9108, which showed faster degradation in pericarp and more accumulation in endosperm.

### Morphology and granules size distribution of caryopsis starch

**Fig. 5:**
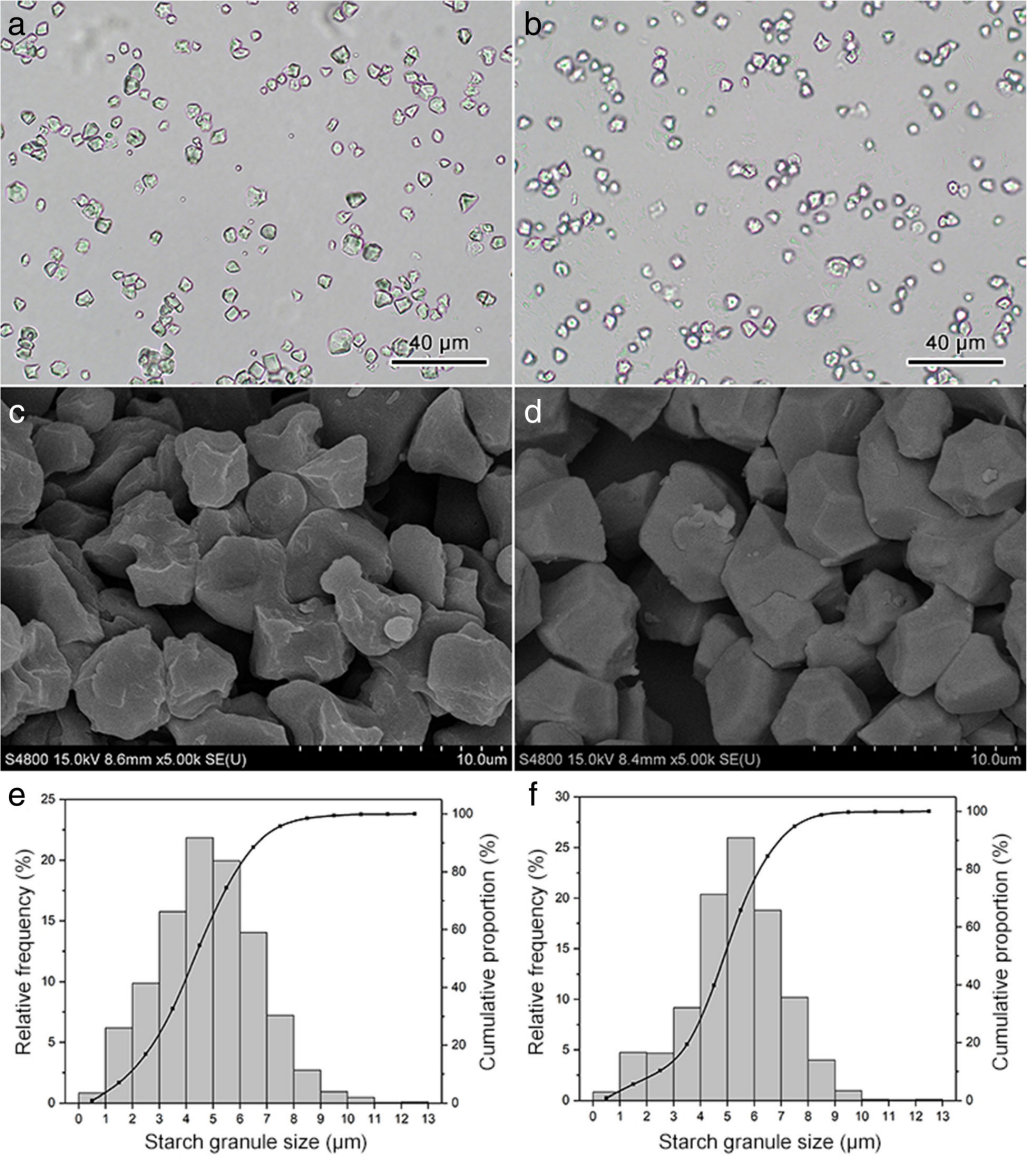
Morphology and granule size distribution of caryopsis starch in two rice cultivars. **a**, **b** micrograph of caryopsis starch in NG9108 and YY2640 under optical microscope; (**c**, **d**) micrograph of caryopsis starch in NG9108 and YY2640 under scanning electron microscope; (**e**, **f**) granules size distribution of caryopsis starch in NG9108 and YY2640

The morphology of starch granules from two rice caryopsis was observed and the granules size distribution was analyzed. Under light microscope, there was no significant difference in the morphology of two rice starch. Most of the starch granules were large and polygonal with irregular shapes, accompanied with some small spherical starch granules (Fig. 5a, b). Under scanning electron microscope, shapes of starch granules of two rice were both polyhedral. The surface of YY2640 starch granules was smooth while the surface of the NG9108 starch granules was uneven and pitted (Fig. 5c, d). The results of two rice starch granules size analysis both presented as an unimodal distribution. The particle size of most starch granules ranged from 2 to 8 μm and the proportion of YY2640 starch granules size in this range was higher than NG9108 (Fig. 5e, f). Although there was no significant difference in the minimum and maximum particle sizes between two rice starch, the average particle size of YY240 starch was significantly larger than that of NG9108 (Table 2).

**Table 2:** Particle size of caryopsis starch in two rice cultivars

cultivar	minimum particle size (μm)	maximum particle size (μm)	average particle size (μm)
NG9108	0.60 ± 0.18	11.41 ± 0.57	4.84 ± 0.15
YY2640	0.77 ± 0.15	11.42 ± 0.50	5.32 ± 0.08^*^

Data are expressed as means ± standard error, *n* = 3. For each column, asterisks indicate significant difference at *p* < 0.05 between rice cultivars as determined by t-test

### Content of amylose and fine structure of caryopsis starch

The amylose content (AC) based on iodine colorimetric analysis is usually defined as apparent amylose content (AAC). The AAC of YY2640 starch was lower than that of NG9108. Due to the absorption of similar wavelengths by amylopectin-iodine and amylose-iodine complexes, AAC is usually an overestimate to the content of amylose. In order to exclude the influence of amylopectin long branch-chains on AAC, the true AC was also determined by assay kit. Although the AC of two rice cultivars exhibited a relatively low level compared with corresponding AAC, its trend of difference (NG9108 higher than YY2640) including the ratio of amylose to amylopectin (Am/Ap) was the same as for AAC (Table 3).

**Table 3:** AAC, AC, Am/Ap and GPC parameters of caryopsis starch in two rice cultivars

cultivar	AAC (%)	AC (%)	Am/Ap	GPC peak area (%)			AP1/AP2
				AP1	AP2	AM	
NG9108	16.99 ± 0.44	12.56 ± 0.62	0.162 ± 0.06	65.36 ± 0.34	21.52 ± 0.15	13.86 ± 0.28	3.04 ± 0.03
YY2640	14.90 ± 0.46^*^	10.21 ± 0.39^*^	0.138 ± 0.13^*^	77.42 ± 0.52^*^	25.68 ± 0.36^*^	11.41 ± 0.21^*^	3.01 ± 0.01

Data are expressed as means ± standard error, *n* = 3. For each column, asterisks indicate significant difference between rice cultivars. AP1, AP2 and AM correspond to short branch chains of amylopectin, long branch chains of amylopectin and amylose, respectively. The AP1/AP2 ratio is determined according to the area ratio of AP1 and AP2 fractions

The molecular weight distributions of starch from caryopsis of two rice cultivars were determined by gel permeation column (GPC). A trimodal distribution with low, middle and high molecular weight peaks was designated as AP1, AP2 and AM fractions, respectively. The area ratios of three fractions were analyzed according to chromatograms and listed in Table 3. The AM fraction represents amylose and the area ratio of AM represents content of amylose. The AC of NG9108 starch determined by GPC was significantly higher than YY2640, which was consistent with the results determined by iodine colorimetric analysis and assay kit. The AP1 and AP2 fractions indicate amylopectin, the AP1 fraction containing short branch-chains of amylopectin and the AP2 fraction consisting of long branch-chains of amylopectin [26]. The area ratios of AP1 and AP2 fractions in YY2640 starch were higher than NG9108, indicating that YY2640 starch contained high levels of amylopectin short and long branch-chains. The area ratio of AP1 to AP2 can be used as an indicator of the extent of amylopectin branching; the higher the ratio, the higher the branching degree [27]. The amylopectin branching degree had no significant difference between two rice starches.

### Crystal structure of caryopsis starch

**Fig. 6:**
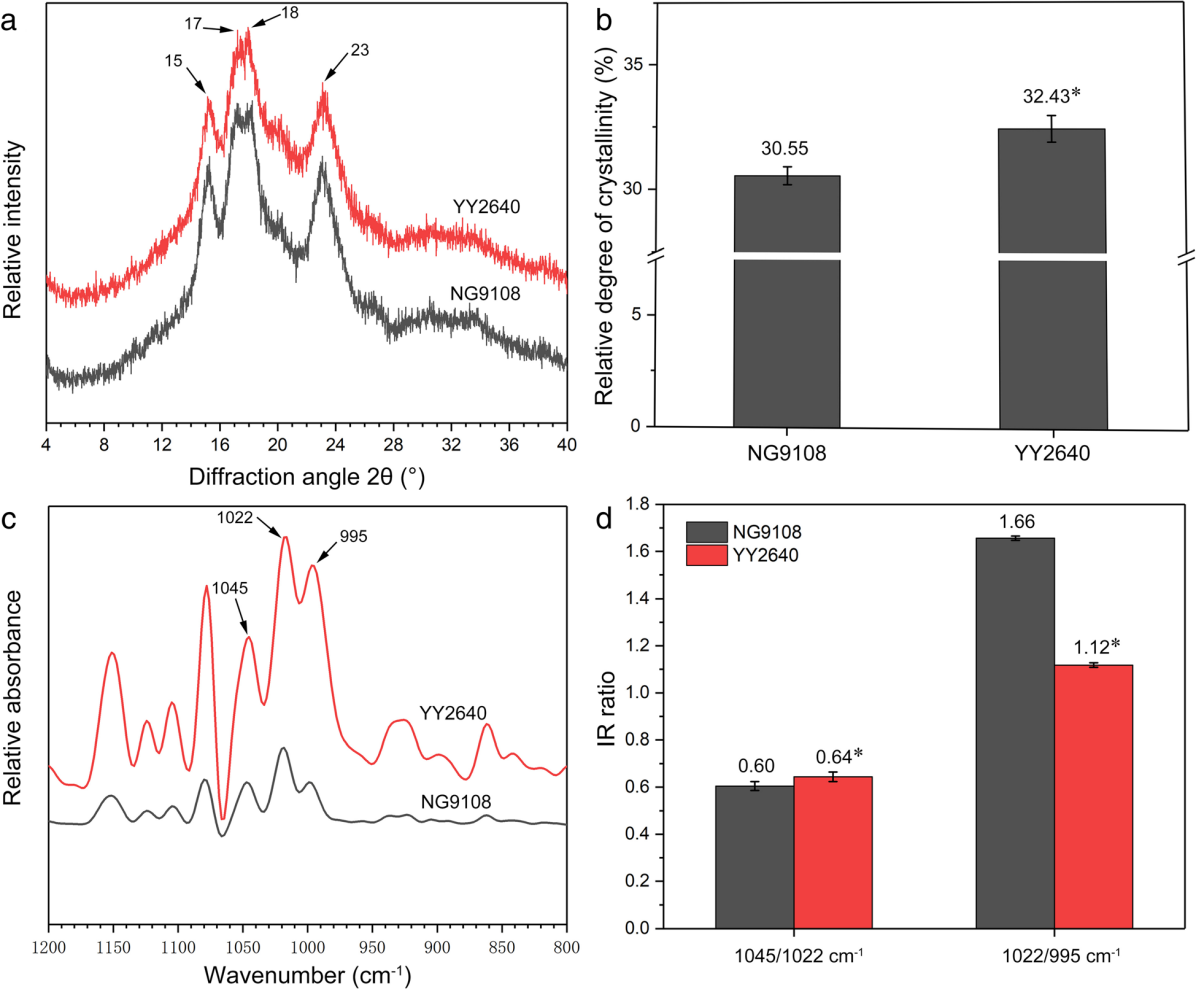
Crystal structure analysis of caryopsis starch in two rice cultivars. **a** XRD spectrum; (**b**) relative crystallinity; (**c**) ATR-FTIR spectrum; (**d**) IR ratio. The arrows in subpanel (**a**) indicate 2θ angles of 15°, 17°, 18° and 23°. The arrows in subpanel (**c**) indicate wave numbers of 995, 1022 and 1045 cm^− 1^. The superscript asterisks indicate significant difference at a *p* < 0.05 level between different cultivars (t-test)

X-ray diffraction (XRD) can be used to detect the crystal structure and qualitatively analyze the crystal type of starch [28]. From the XRD spectrum, the starch of the two rice cultivars both had the characteristics of A-type crystal with strong diffraction peaks at about 2θ of 15° and 23°, and an unresolved doublet at around 2θ of 17° and 18°, which was consistent with the crystal characteristics of normal cereal starch (Fig. 6a). Although there was no significant difference in the XRD spectrum of starch from two rice caryopsis, the relative crystallinity of YY2640 starch was higher than that of NG9108 (Fig. 6b). This may be related to the size of starch granules, and the starch of YY2640 with larger granules size had higher relative crystallinity.

Attenuated total reflectance-Fourier transform infrared (ATR-FTIR) spectroscopy can be used to study the external ordered structure of starch granules and the intensity of absorbance at 1045, 1022 and 995 cm^− 1^ varies when starch conformation changes [29]. The band at 1045 cm^− 1^ is the characteristic structure of crystalline regions in starch, the band at 1022 cm^− 1^ is sensitive to the amorphous region in starch granules, and the band at 955 cm^− 1^ is related to the bonding in hydrated carbohydrate helices [30]. Therefore, the ratio of 1045/1022 cm^− 1^ is regarded as a measure to quantify the ordered degree of starch external structure and the ratio of 1022/995 cm^− 1^ can be used as an index of the proportion of amorphous to ordered carbohydrate structure in starch. As shown in Fig. 6c, the starch of the two rice exhibited similar ATR-FTIR spectra, indicating that their types of crystal were similar (both A-type crystal), which was in conformity with the XRD results. However, there were significant differences in the IR ratios of 1045/1022 cm^− 1^ and 1022/995 cm^− 1^ between two rice starch (Fig. 6d). Compared with NG9108, the ratio of 1045/1022 cm^− 1^ was higher and the ratio of 1022/995 cm^− 1^ was lower in YY2640. The data indicated that YY2640 starch had the higher external ordered degree, which may be caused by the granules size and relative crystallinity of starch.

### Pasting properties of caryopsis starch

The pasting properties of rice starch were determined by rapid viscosity analyzer (RVA) and the data is shown in Table 4. There were significant differences in peak viscosity, trough viscosity, breakdown value, setback value, pasting temperature and pasting time of the two rice starch except final viscosity. Pasting temperature is the temperature when the viscosity of starch paste begins to rise. Compared with NG9108, the pasting temperature of YY2640 starch was lower and the pasting time was shorter. Peak viscosity is the maximum viscosity of starch paste during heating with water, trough viscosity is the viscosity at the end of constant temperature phase and final viscosity is a stable viscosity of starch paste after cooling. The peak viscosity, trough viscosity and final viscosity of YY2640 starch were significantly higher than those of NG9108, indicating that YY2640 starch had larger degree of swelling and higher stability of swollen granule structure. Breakdown value shows the thermal stability and shear resistance of starch paste while setback value shows the retrogradation tendency of starch paste. The breakdown value of YY2640 starch was higher but the setback value was lower than NG9108, which indicated that the rice of YY2640 neither became stiff nor retrograded during cooking. The above results showed that YY2640 starch was easier to gelatinize and the starch paste was more stable after gelatinization, thus the rice of YY2640 might have the potential of good cooking and eating quality.

**Table 4:** Pasting properties of caryopsis starch in two rice cultivars

cultivar	PV (cP)	TV (cP)	BD (cP)	FV (cP)	SB (cP)	P_temp_ (°C)	PT (min)
NG9108	2734 ± 125	763 ± 20	1971 ± 106	1447 ± 37	684 ± 91	78.10 ± 1.30	4.44 ± 0.11
YY2640	3657 ± 137^*^	972 ± 41^*^	2685 ± 120^*^	1443 ± 39	471 ± 84^*^	73.45 ± 0.95^*^	3.58 ± 0.21^*^

Data are shown as means ± standard error, *n* = 3. For each column, asterisks indicate significant difference between rice cultivars at *p* < 0.05 (t-test)

PV, peak viscosity; TV, trough viscosity; BD, breakdown value; FV, final viscosity; SB, setback value; P_temp_, pasting temperature; PT, pasting time. Breakdown value = peak viscosity - trough viscosity, setback value = final viscosity - trough viscosity

## Discussion

Lodging is one of the important factors limiting the high yield of rice and the lodging resistance is mainly determined by plant height. The plant height is closely related to biological yield and the appropriate increase in plant height may lead to the increase of biomass, which is the material basis for the increase in kernel number and 1000-grain weight. Additionally, the thick degree of stalk and the amount of storage substance in stem-sheath can also affect the ability of resisting to lodging and play an important role on improving the seed setting rate and grain weight [31]. Previous studied revealed the relationship between large panicle, physiological properties and super-high-yield in Yongyou series rice cultivar and found its outstanding physiological properties with strong source, efficient flow and great sink [6]. Therefore, the ideal plant type of YY2640 with large panicle, high and thick stems may lead to its harmonious source-translocation-sink characteristic and promote the increase of biomass accumulation in gains, which ultimately resulted in good gains characteristics and high yield.

The base of caryopsis is thick and the upper part is thin in appearance. Then the upper part gradually becomes obtuse and smooth as the growth of caryopsis, which is basically as wide as the base part. Previous studies have found that the shape of caryopsis has a certain correlation with the quality of rice [32]. The slender shaped caryopsis possesses a good appearance quality with less chalkiness and high transparency, which is also observed in this study. With the development of caryopsis, the grain weight increased and the storage substances enriched. The substance accumulation in caryopsis is actually the transportation of photoassimilates from leaf (source) to grain (sink) through transfer tissue. Therefore, sink strength is an important indicator determining grain weight. Sink strength can reflect the ability of caryopsis to accept and transform assimilates, determined by sink capacity and sink activity. Sink capacity is related to the size of caryopsis while sink activity is linked with caryopsis growth and respiration rate [33]. In this study, the caryopsis of YY2640 was large in size at early development stage and the rate of grain filling was fast, indicating high sink capacity and strong sink activity. This may promote the growth of YY640 caryopsis and the increase of grain weight, eventually resulting in the higher fresh and dry weight of caryopsis compared with NG9108.

The amyloplast in caryopsis is mainly divided into two types, namely pericarp amyloplast and endosperm amyloplast, which play different roles in the development of caryopsis. The pericarp amyloplast is a temporary energy storage that gradually degrades to provide energy for the growth of pericarp, while the endosperm amyloplast is the ultimate energy storage which reserves nutrient for the filial development [34, 35]. The development of pericarp and pericarp amyloplast affects the growth and shape of caryopsis and the poor growth of pericarp can result in the maldevelopment of caryopsis. According to the microstructure, the apoptosis of pericarp cells and degradation of amyloplast began at 5 DAA. Furthermore, the pericarp amyloplast in YY2640 caryopsis degraded faster to provided energy and nutrition for the growth of pericarp and endosperm, which promoted the rapid development and grain filling of YY2640 caryopsis. Therefore, it can be speculated that the development of pericarp amyloplast is compatible with the growth of caryopsis, which is consistent with the results of Wang et al. [36]. The development of endosperm amyloplast is linked with the input pathway of storage substance in caryopsis. Yu et al. proposed a possible pathway of nutrient transfer in wheat caryopsis [37]. Nutrients from the vascular bundle are first unloaded into nucellar projection tissue via chalaza and then shunted in the apoplast outside nucellar projection. Some of the nutrients are transported directly to endosperm by transfer cells. Another part of the nutrients is transported toward the periphery of endosperm by apoplast and then into endosperm through aleurone layer cells. This similar nutrient transfer pathway in rice caryopsis may be responsible for good endosperm enrichment and amyloplast development of YY2640. The caryopsis of YY2640 was slender shaped and the distance between inner endosperm cells and aleurone layer was relatively close. Thus the nutrient transport distance was short, which was conducive to the enrichment of endosperm amyloplast. On the contrary, the shape of NG9108 caryopsis was broad and thick, and the nutrients required for the formation of amyloplast were transported to endosperm for a long distance, resulting in poor enrichment of endosperm amyloplast. In addition, the activity of starch metabolism-related enzymes such as soluble starch synthase (SSS), granule-bound starch synthase (GBSS) has a direct impact on starch biosynthesis and amyloplast formation [38]. The difference in the development of amyloplast between two rice may be due to the relevant genes’ differential expression and activity of starch metabolism enzyme. In a word, the formation of rice quality is involved with various physiological and biochemical processes such as synthesis and transport of photosynthetic products, regulation of key enzyme and so on. YY2640 had an ideal plant type, whose caryopsis grew fast with appropriate shape. Also, the endosperm of YY2640 caryopsis was well filled resulting from good development of amyloplast. All of these might lead to the good quality of YY2640 caryopsis.

The physicochemical properties of starch such as particle size, morphology, crystal structure and external ordered degree are important factors affecting the functional properties of starch, that are closely related to the nutrition and processing quality of starch [39], thus determining the quality of rice. For good quality rice varieties, the starch granules are polyhedral and aligned in size, while the starch granules of poor quality rice varieties are polygonal with a rough surface and loose arranged [40]. In this study, YY2640 starch granules had a regular polyhedral shape with smooth surface, while the surface of NG9108 starch granules were rough and pitted. Meanwhile, the results of granules size analysis showed that the size of YY2640 starch granules was larger than NG9108. Thus, it can be seen from the starch morphology that YY2640 may have the potential of good starch quality. Crystallinity is an important parameter for characterizing the crystal structure of starch, which directly influences starch physicochemical properties [41]. Previous studies have shown that the relative crystallinity of starch is affected by the size of starch granules [42]. YY2640 starch granules were larger in size, thus exhibiting higher relative crystallinity in this study, which is in accordance with previous report [43]. The reason for this result may be the amylose content difference and the chain length distribution of amylopectin [44]. The content of amylose and the branching of amylopectin play a decisive role in the formation of starch crystal structure. Amylose distributed in the crystal layer of starch granules will reduce the stability of crystal structure, while the short branch of amylopectin can provide a higher external ordered degree for starch granules [45]. The difference in the composition and proportion of amylose and amylopectin between YY2640 and NG9108 starch affected the morphology of starch granules to some extent, resulting in different functional properties.

The pasting of starch is the basis of starch utilization. During the pasting process, starch granules absorb water to irreversibly swell and eventually rupture. The crystal structure of starch is destroyed, showing unique physical properties. Therefore, pasting properties, as an important indicator of starch quality, are of great significance for the processing and utilization of starch [46]. The pasting properties of starch are affected by many factors, involved with composition of starch (amylose/amylopectin ratio, protein and lipid content), structure of amylopectin molecules (unit chain length, branching degree), granules structure, granules morphology, granules size and distribution [47]. Among them, amylose content and amylopectin structure are the main factors affecting starch pasting properties, which ultimately determine the cooking and eating quality of rice. Amylose can inhibit the swelling of starch granules, while the chain length distribution of amylopectin and the molecular weight of amylose have a synergistic effect on starch pasting [48]. Generally, the higher content of amylose in rice starch, the higher pasting temperature, higher setback value and lower peak viscosity the starch possesses. Thus, the cooked rice has the feature of low stickness, hard texture, dim color and poor taste [49]. Conversely, rice starch with low amylose content is easy to be gelatinized and has a high breakdown value. The rice is soft and sticky with a good taste after cooking [50]. The YY2640 starch showed better pasting properties compared with NG9108 due to its lower amylose content. This may result in the potential of good rice quality for YY2640. In addition to the amylose content, however, the ratio of long-chain branches and short-chain branches of amylopectin also affects the taste quality of rice. Therefore, the evaluation of YY2640 rice quality needs further experiments on more starch properties and indexes of cooking and eating quality, such as protein content, lipid content, gel consistency, texture properties and so on.

## Conclusions

In this study, structural characteristics of amyloplast development in rice caryopsis were observed. The development of caryopsis amyloplast in YY2640 was better than NG9108, showing faster degradation rate of pericarp amyloplast and better filling degree of endosperm amyloplast. Meanwhile, physicochemical properties of rice starch were investigated and their relationship with structural development of amyloplast was discussed. YY2640 starch showed better pasting properties with lower pasting temperature, higher peak viscosity and lower setback value because of lower apparent amylose content. The results indicated that better development and filling of amyloplast in YY2640 might lead to better starch properties. Thus YY2640 may have the potential of good rice quality.

## Methods

### Plant materials

**Fig. 7:**
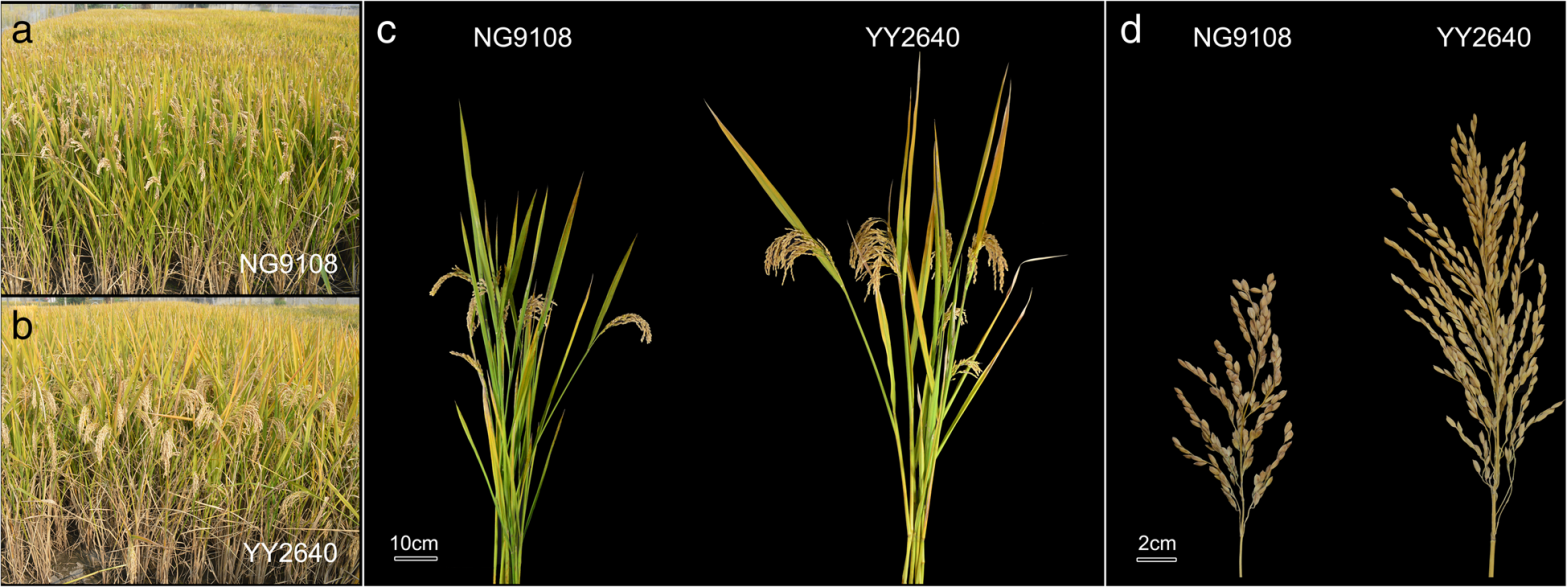
Growth and profile of two rice plant. **a**, **b** field growth; (**c**) plant type; (**d**) panicle morphology. Scale bar: (**c**) 10 cm, (**d**) 2 cm

The rice cultivars selected in this study were YY2640 and NG9108 which were provided by Ningbo Academy of Agricultural Science and Jiangsu Academy of Agricultural Sciences respectively. YY2640 and NG9108 are both super hybrid rice varieties with the characteristics of high yield and widely cultivated in the middle and lower reaches of the Yangtze River. YY2640 is a typical large panicle typed super rice while NG9108 has a relatively normal type of panicle. Compared with NG9108, YY2640 has a good growth in the field and its plant type is high and strong with large panicle and great number of grains (Fig. 7). The rice was planted in the experimental field of the Crop Cultivation Key Laboratory of Yangzhou University, Jiangsu Province, China in 2017 during rice cropping season. The crop previous to rice was wheat and the soil was sandy loam. The soil parameters were as follows: organic matter content 0.86%, total nitrogen 1.4 g/kg, available phosphorus 35.1 mg/kg, available potassium 88.3 mg/kg, pH of 8.01. During rice flowering, individual florets were marked with a marking pen and tagged to label the anthesis date.

### Caryopsis morphological observation, histochemical staining and growth index determination

Caryopses at different DAA were cut transversely from center and subsequently stained with I_2_/KI solution at 25 °C for 30 s. All caryopses and their corresponding transections were photographed under a stereomicroscope (MZ6, Leica, Germany) equipped with a digital camera. Meanwhile, the caryopses at different DAA were collected to determine fresh weight and dried in a fan-forced oven at 105 °C for 1 h to deactivate enzymes and then at 42 °C to attain constant weights for dry weight determination. Water content was calculated according to the formula below: water content = (fresh weight - dry weight)/fresh weight.

### Panicle traits determination

Mature rice was harvested to determine panicle length, panicle weight, grain number per panicle, seed setting rate and 1000-grain weight.

### Microstructure of caryopsis observation

Caryopses at different development stage were collected and cut transversely into 2 mm thin slices from the middle with a razor blade and immediately immersed in 2.5% glutaraldehyde fixative at 4 °C for 4 h. Then, the samples were rinsed thrice with 0.1 M phosphate buffer (pH 7.2) and dehydrated in a graded ethanol series followed by propylene oxide replacement. Afterward, the samples were infiltrated and embedded in Spurr’s resin and polymerized at 70 °C for 12 h. Eventually, the samples were cut into 1 μm thickness slices using an ultramicrotome (Ultracut R, Leica, Germany), pasted on glass slides, stained with 1% toluidine blue for 5 min, rinsed, dried, observed under a light microscope (DMLS, Leica, Solms, Germany) and photographed by a camera (TrueChromeII, Tucsen, China). Each rice cultivar selected three caryopses as repeats, each of which was from different rice panicles.

### Numbers and areas of amyloplast measurement and calculation

According to the method previously described by Chen et al. [51], Photoshop and Image-Pro Plus were used to count numbers of amyloplast and calculate relative areas of amyloplast based on micrographs. (3 replicate caryopses and 10 micrographs each caryopsis).

### Starch of caryopsis isolation

The mature caryopses were placed in a mortar and fully ground to homogenate with water. The homogenate was filtered through eight layers of gauze and a 100-mesh sieve. The filtrate was centrifuged at 3500 *g* for 5 min, and then the supernatant was poured. The remaining precipitate was resuspended with 0.2% NaOH solution and the suspension was centrifuged at 3500 *g* for 10 min. The supernatant was discarded and the upper colored layer was scraped off. The starch was collected until the supernatant was clarified and dried in a 40 °C oven to constant weight. The dried starch was thoroughly ground into powder using a mortar and collected with a 100-mesh sieve.

### Content of amylose determination

The determination of AAC referred to the method of Man et al. [52]. Starch was defatted using 85% methanol and then dissolved in dimethyl sulphoxide containing urea solution. The iodine absorption spectrum was scanned from 400 to 900 nm using a spectrophotometer (Ultrospec 6300pro, Amershan Biosciences, UK). The AAC was calculated from absorbance at 620 nm by reference to a standard curve. The true AC was measured using Megazyme Amylose/Amylopectin Assay Kit (K-AMYL) according to the manufacturer’s instructions. The amylose/amylopectin ratio were calculated based on AC.

### Morphology of starch granules observation and granules size distribution analysis

About 1 mg of dried starch and 500 μL of anhydrous ethanol were mixed and starch ethanol solution (20 μL) was pipetted onto a sample table filmed with aluminum foil and dried at 40 °C for 1 h. After the surface was plated with gold by a vacuum ion sputtering apparatus (EM SCD500, Leica, Germany), the samples were photographed under a field-emission scanning electron microscope (S4800, Hitachi, Japan). A small amount of dried starch was scattered by 50% glycerol, and then pipetted onto a glass slide covered with a coverslip. The freely dispersible sample was photographed under a light microscope. Image-Pro Plus was used to measure the maximum axial length of starch granules in the image. The granule size distribution was plotted according to the percentage of starch granules in each granule size range. Each starch sample was randomly counted for 2000 starch granules and the measurement repeated thrice.

### GPC analysis

The starch samples were deproteinized with protease and sodium bisulfite and debranched with isoamylase (EC3.2.1.68, E-ISAMY, Megazyme) according to the method previously described [53]. The molecular weight distribution of debranched starch was analyzed using a PL-GPC 220 high-temperature chromatograph system (Agilent Technologies UK Limited, Shropshire, UK) with three columns (PL110–6100, 6300, 6525) and a differential refractive index detector following the method of Zhang et al. [54].

### XRD analysis

About 200 mg of dried starch sample was placed in the groove of quartz sample table and flattened with a glass slide. The sample was scanned on an X-ray diffractometer (D8 Advance, Bruker, Germany) from the diffraction angle (2θ) ranged at 4° to 40° with a count time of 0.6 s to obtain an XRD spectrum. Photoshop and Image-Pro Plus software were used to analyze the relative crystallinity according to the method of Nara and Komiya [55] with minor modifications.

### Structural order of starch external region analysis

30 mg of dry starch and 25 μL of ultrapure water were mixed in a centrifuge tube and the suspension was dropped on the probe of attenuated total reflection mode sample table equipped on Fourier transform infrared spectrometer (7000, Varian, USA). The spectrum of sample from 1200 cm^− 1^ to 800 cm^− 1^ was recorded followed by deconvolution. The deconvolution enhancement factor was 1.9 and the half-band width was 19 cm^− 1^. The intensity of bands at 1045, 1022 and 995 cm^− 1^ was measured and the ratio of 1045/1022 cm^− 1^ and 1022/995 cm^− 1^ was calculated using Image-Pro Plus.

### Pasting properties determination

The pasting properties of starch were determined using RVA (Model 3D, Newport Scientific, Australia) according to the method previously reported [56]. 3 g starch and 25 g ultrapure water were mixed in an aluminum crucible and tested on the machine. Starch samples were heated from 50 °C to 95 °C at 12 °C/min, maintained at 95 °C for 2.5 min, cooled to 50 °C at 12 °C/min and finally held at 50 °C for 1.4 min. The rotor speed is 160 r/min. Each sample measured three replicates.

### Statistics analysis

The experimental data was recorded using Microsoft Excel and significance test was performed using t-test at a probability significance level of *P* < 0.05 in SPSS. Origin and Photoshop were used to plot the figures.

## Data Availability

All data generated or analyzed during this study are included in this manuscript.

## Abbreviations

AAC: apparent amylose content
AC: amylose content; Am: amylose
Ap: amylopectin
ATR-FTIR: attenuated total reflectance-Fourier transform infrared
DAA: days after anthesis
GBSS: granule-bound starch synthase
GPC: gel permeation column
NG9108: Nangeng9108
RVA: rapid viscosity analyzer
SSS: soluble starch synthase
XRD: X-ray diffraction
YY2640: Yongyou2640
